# The assessment of left ventricular mechanical dyssynchrony from gated ^99m^Tc-tetrofosmin SPECT and gated ^18^F-FDG PET by QGS: a comparative study

**DOI:** 10.1007/s12350-021-02737-0

**Published:** 2021-07-19

**Authors:** Sebastian Lehner, Frank Philipp Graner, Maximilian Fischer, Harun Ilhan, Peter Bartenstein, Andrei Todica

**Affiliations:** 1 Department of Nuclear Medicine, University Hospital, Ludwig-Maximilians-Universität, Marchioninistraße 15, 81377 Munich, Germany; 2 Ambulatory Health Care Center Dr. Neumaier & Colleagues, Radiology, Nuclear Medicine, Radiation Therapy, Bahnhofstraße 24, 93047 Regensburg, Germany; 3 Department of Internal Medicine, Cardiology, University Hospital, Ludwig-Maximilians-Universität, Munich, Germany

**Keywords:** Gated SPECT, gated PET, left ventricular dyssynchrony, phase analysis, QGS

## Abstract

**Background:**

Due to partly conflicting studies, further research is warranted with the QGS software package, with regard to the performance of gated FDG PET phase analysis as compared to gated MPS as well as the establishment of possible cut-off values for FDG PET to define dyssynchrony.

**Methods:**

Gated MPS and gated FDG PET datasets of 93 patients were analyzed with the QGS software. BW, Phase SD, and Entropy were calculated and compared between the methods. The performance of gated PET to identify dyssynchrony was measured against SPECT as reference standard. ROC analysis was performed to identify the best discriminator of dyssynchrony and to define cut-off values.

**Results:**

BW and Phase SD differed significantly between the SPECT and PET. There was no significant difference in Entropy with a high linear correlation between methods.

There was only moderate agreement between SPECT and PET to identify dyssynchrony. Entropy was the best single PET parameter to predict dyssynchrony with a cut-off point at 62%.

**Conclusion:**

Gated MPS and gated FDG PET can assess LVMD. The methods cannot be used interchangeably. Establishing reference ranges and cut-off values is difficult due to the lack of an external gold standard. Further prospective research is necessary.

**Supplementary Information:**

The online version contains supplementary material available at 10.1007/s12350-021-02737-0.

## Background

The concept of measuring left ventricular mechanical dyssynchrony (LVMD) in heart failure (HF) patients is compelling for its potential use as a tool to select candidates for cardiac resynchronization therapy (CRT) and monitor the outcome.^1^,^2^ While lifesaving in many cases, CRT has unfortunately been prone to a high failure rate of up to 30%, which might be partly explained by the limited amounts of underlying LVMD.^2^,^3^ Phase analysis might help to ameliorate this situation. Other use cases for phase analysis include the assessment of dyssynchrony in coronary artery disease and the assessment of diastolic left ventricular dyssynchrony.^1^,^3^

Gated myocardial perfusion single-photon emission computed tomography (MPS) and, in recent times, gated ^18^F-fluorodeoxyglucose positron emission computed tomography (FDG PET) are non-invasive tools that offer simplicity, high reproducibility, and wide availability for the assessment and monitoring of LVMD.^1^

Since its inception in the year 2005, phase analysis of gated MPS datasets has been implemented into all major commercial software packages, such as Quantitative Gated SPECT, the Emory Cardiac Toolbox, or 4DM SPECT, and recently been extended to gated PET datasets.^1^,^3^

Even though the method has been in use for the better part of 15 years, the amount of available evidence is still limited. Most of the evidence comprises gated SPECT. Data for gated PET have been added.

As of yet, it remains unclear which parameters best measure dyssynchrony, which normal values define synchrony (and to this effect dyssynchrony), and which parameters best predict dyssynchrony in otherwise healthy individuals and HF patients alike. There is a wide variation in numeric evidence between software packages and methods.^4^

In recent years, several groups tried to define normal values for gated SPECT studies using different software packages. One of the most comprehensive of such studies was published in 2018 by Hämäläinen et al.^5^ The study aggregated a plethora of reference ranges for many different functional gated MPS values from a rigorously selected normative cohort using the QGS software package. Not many attempts have been made to define phase analysis reference or cut-off values for gated PET. Two studies were published in 2011 and in 2012, both used the Rubidium tracer and employed the Emory Cardiac Toolbox and 4DM SPECT, respectively.^3^

Other studies evaluated the diagnostic performance of gated PET as compared to gated SPECT.

In 2011 Pazhenkottil et al.^6^ used the Emory Cardiac Toolbox to compare the diagnostic performance of FDG-PET vs MPS. However, only the parameters BW and Phase SD were analyzed. Furthermore, the used cut-off values to divide the cohort into synchronous and dyssynchronous patients were originally tailored to predict the response following CRT.

In 2013 Wang et al.^7^ used QGS to compare phase analysis with FDG-PET and MPS and tried to identify confounders like left ventricular remodeling or poor FDG uptake. However, likewise only the parameters BW and Phase SD were evaluated and no attempt was made to identify dyssynchronous patients to assess the diagnostic performance of FDG-PET or perform an ROC analysis to define possible PET cut-off values.

In 2020 Tian et al.^8^ used QGS to compare phase analysis with FDG-PET and MPS. They evaluated BW, Phase SD, and Entropy. However, the used cut-off values to divide the cohort into synchronous and dyssynchronous patients were not specific for the QGS software and partly tailored to predict the response following CRT. No ROC analysis was performed to define possible PET cut-off values for the detection of dyssynchrony.

To make a valuable contribution to this existing body of evidence, the following topics were investigated in our study:

- Feasibility of retrospective phase analysis of gated MPS and gated FDG-PET datasets using the QGS software package with a comprehensive comparison of the phase analysis parameters Bandwidth (BW), Phase Standard Deviation (Phase SD), and Entropy between SPECT and PET datasets to validate existing evidence.
- Evaluation of the diagnostic performance of gated FDG PET as compared to gated MPS as the reference standard based on reference values specifically established for SPECT datasets, and the QGS software package and calculation of specific gated FDG-PET cut-off values for the parameters BW, Phase SD, and Entropy to optimize diagnostic performance, which to the knowledge of the authors has not been done before.

## Materials and Methods

### Study Population

Our study population consisted of 93 consecutive patients (83 male, mean age 65 ± 11 years, mean weight 87 ± 22 kg, SPECT left ventricular ejection fraction 34% ± 15%, PET left ventricular ejection fraction 33% ± 15%, mean injected ^99m^Tc-tetrofosmin/^99m^Tc-MIBI dose 425 ± 152 MBq, mean injected FDG dose 268 ± 50 MBq). For baseline characteristics see Table 1.

**Table 1:** Baseline characteristics of the study population.

Baseline characteristics (n = 93)	
Gender (m, f)	83 male
Age (years)	64 ± 11
Weight (kg)	87 ± 22
Dose SPECT (MBq)	425 ± 152
Dose PET (MBq)	268 ± 50
TPD (%)	23 ± 15
Mismatch (%)	6.3 ± 6.3
Scar (%)	16 ± 13
SPECT EDV (mL)	214 ± 103
SPECT ESV (mL)	154 ± 93
SPECT LVEF (%)	34 ± 15
PET EDV (mL)	214 ± 94
PET ESV (mL)	154 ± 91
PET LVEF (%)	33 ± 15

Patients with a history of coronary artery disease were referred to our institution for the assessment of myocardial viability.

An integrated SPECT/CT scanner and a dedicated triple-head SPECT camera system were used for routine MPS under resting conditions. An FDG PET/CT scan was performed on average 17 days after the MPS scan on dedicated PET/CT systems.

This retrospective study was conducted with the approval of the local ethics committee (Ethikkommission der Medizinischen Fakultät der LMU München).

### SPECT Imaging

For rest MPS ^99m^Tc-tetrofosmin was administered intravenously and SPECT scans were commenced 30-45 minutes after the application of the tracer. One camera used in the study, was a dual-head hybrid SPECT/CT camera (Symbia, Siemens Medical Systems, Erlangen, Germany) with a parallel-hole LEHR collimator. The energy window was centered at 140 keV ± 20%; the two detector heads were set at an angle of 90° and covered an arc of 180° at 64 rotational steps. Each single projection lasted 23 seconds. An electrocardiogram R-wave detector was employed for ECG-gating; 12 emission frames were recorded during each cardiac cycle. While images were reconstructed as static and as gated perfusion images, only the gated images were used in the current study, and the static images were used for clinical reporting.

The second camera used was triple-head SPECT camera system (Prism 3000 XP; Philips/InterMedical, formerly Picker, Cleveland, OH). It was equipped with a parallel-hole LEHR collimator. The symmetrical 20% energy window was likewise centered at 140 keV. The three detector heads were set at a 120° angle and performed a 360° rotation with 20 steps per head, each step lasting 60 seconds. ECG-gating was performed as described above. Images were again reconstructed as gated and static images; static images were used for clinical reporting, and gated images were analyzed in the present study.

### PET/CT Imaging

Cardiac FDG PET/CT was performed on 64-slice CT PET/CT systems (Biograph 64, Siemens Medical Systems, Erlangen, Germany and GE Discovery 690, GE Healthcare, Chicago, USA). Patients received 250 mg Acipimox 120 minutes before the scan, and FDG was administered 30 minutes before the scan.

Non-diabetic patients received an oral glucose load, and diabetic patients were treated according to a modified protocol of the American Society of Nuclear Cardiology.^9^ 60 minutes prior to the PET scan, diabetic patients received a light meal and their regular insulin dose. An intravenous catheter was placed into each cubital vein for the injection of glucose and the measurement of blood glucose levels. Over the course of 8 minutes, two syringes filled with 20% glucose solution (.2 g per kg bodyweight), one with additional insulin (.2 dose units per kg bodyweight), were injected. Blood glucose levels were closely monitored. After glucose levels had peaked and started to decline, the FDG was injected. 30 minutes later the patients were placed inside the scanner and a low-dose spiral CT (120 keV, 11 mAs) was acquired for attenuation correction, this was followed by a 20-minute emission scan with ECG-gating. The PET images were reconstructed as static and gated images (matrix size 168 x 168, zoom factor 1). Static images were used for clinical reporting, and gated images were analyzed in the present study.

### Image Analysis

**Figure 1:**
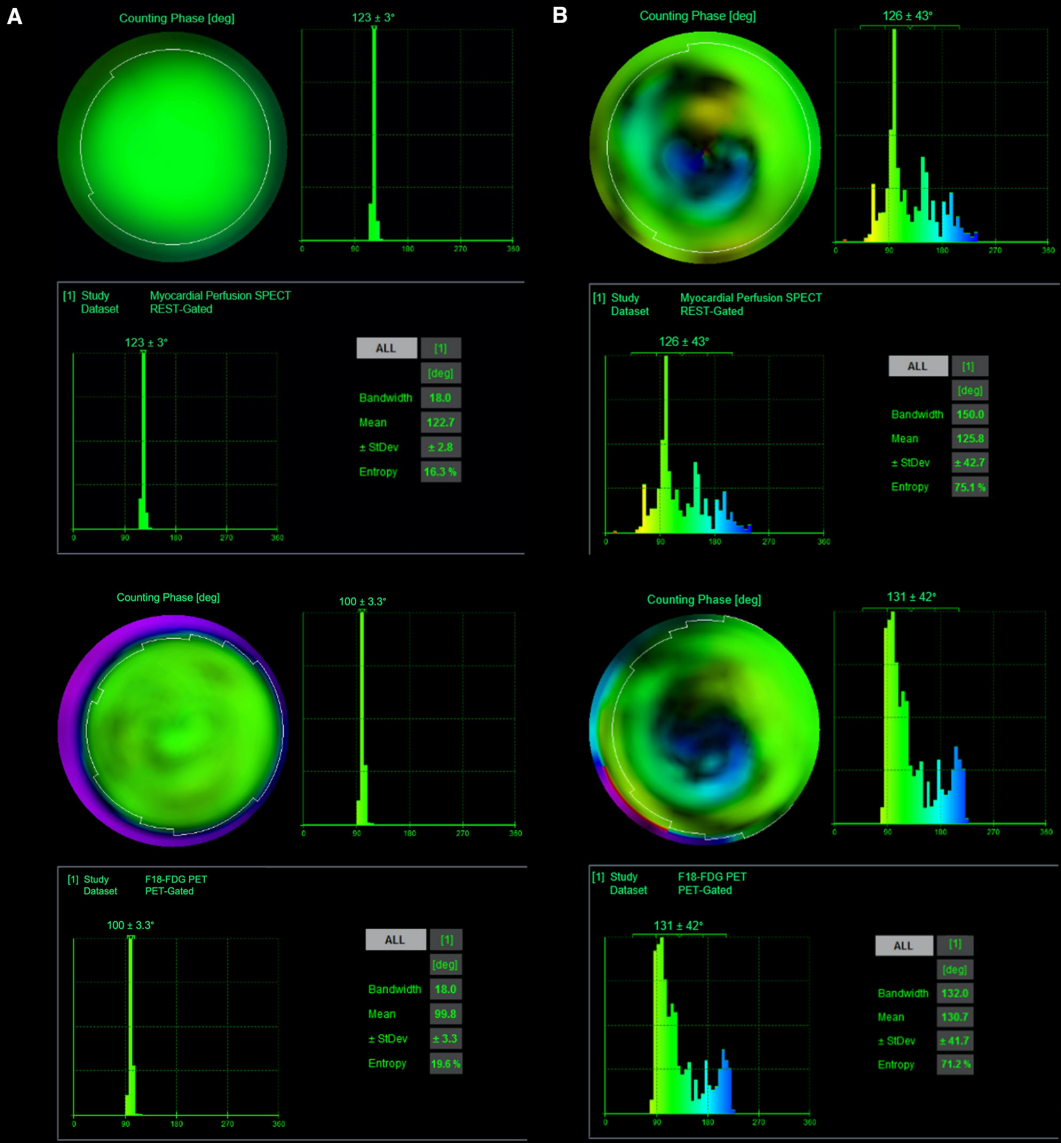
Phase analysis with QGS. **A** shows the SPECT (upper image) and PET (lower image) phase analysis of the same patient without dyssynchrony. **B** shows the SPECT (upper image) and PET (lower image) phase analysis of the same patient with dyssynchrony

Gated MPS and gated FDG PET datasets were analyzed with the Quantitative Gated SPECT software (QGS, Cedars-Sinai, Los Angeles, California) as described before.^5^ In brief, the datasets were loaded into the software, and the myocardium was delineated by an automatic algorithm. Optimal delineation was visually verified, and minor manual adjustments were made, if necessary. The parameters BW, Phase SD, and Entropy were calculated and displayed by the software, representing of the amount of myocardial dyssynchrony (Figure 1). Gated SPECT served as the reference standard and patients were diagnosed as synchronous or dyssynchronous based on published QGS rest reference values for BW (17.0-63.7°), Phase SD (4.4-26.5°), and Entropy (44.0%-63.7%).^5^ Patients were assigned to the dyssynchronous cohort, when values for all three phase analysis parameters were pathological. Otherwise, they were assigned to the synchronous group. In a second analysis, patients were assigned to the dyssynchronous cohort, if two out of the three phase analysis parameters were pathological. Otherwise, they were assigned to the synchronous group.

### Statistical Analysis

All variables are reported as mean ± standard deviation (SD).

Statistics were calculated with the commercial statistics software Wizard 2 (Version 2.0.4 (250), Evan Miller).

The Shapiro–Wilk test was used to test for normal distribution.

The Wilcoxon signed-rank test was used to test for differences in two groups with repeated measurements.

The Mann–Whitney *U* test was used to test for differences between two groups that were not normally distributed.

Pearson’s r was calculated as a measure of linear correlation between two datasets; scatter diagrams and Bland–Altman plots were used for visualization and further analysis.

Kappa was calculated as a measure of agreement between SPECT and PET.

Youden’s *J* statistic was used to calculate cut-off values optimized for sensitivity and specificity in ROC analysis. A *z* test was used to compare the AUC.

*P* values < .05 were considered statistically significant.

## Results

The gated MPS scans as well as the gated FDG PET scans could successfully be analyzed using the QGS software.

Gated MPS revealed a mean BW of 94 ± 55°, a mean Phase SD 26 ± 16°, and a mean Entropy of 58% ± 15%.

Gated FDG PET revealed a mean BW of 104 ± 53°, a mean Phase SD of 30 ± 17°, and a mean Entropy of 58% ± 15%.

Mean BW and mean Phase SD were significantly different between gated MPS and gated FDG PET as shown in Table 2.

**Table 2:** BW and Phase SD differed significantly between SPECT and PET.

	SPECT	PET	p
BW (°)	94 ± 55	104 ± 53	.022
Phase SD (°)	26 ± 16	30 ± 17	.004
Entropy (%)	58 ± 15	58 ± 15	.601

BW showed only a moderate correlation between SPECT and PET (*R* = .55, *P* < .001).

Phase SD likewise showed only a moderate correlation between SPECT and PET (*R* = .58, *P* < .001).

Entropy showed a high correlation between SPECT and PET (*R* = .73, *P* < .001).

**Figure 2:**
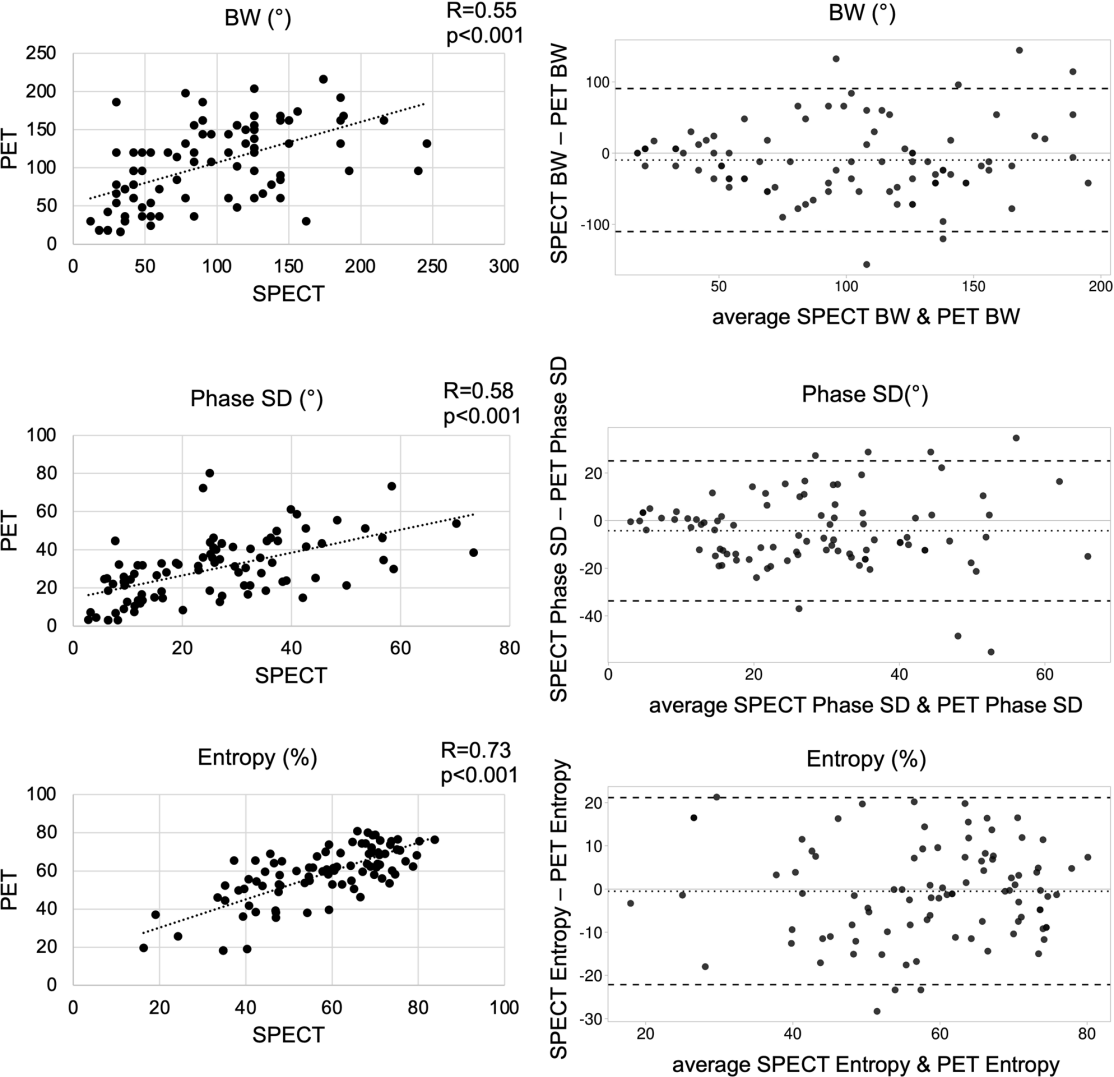
Correlation of BW, Phase SD, and Entropy between PET and SPECT

All parameters showed satisfactory agreement on Bland–Altman plots (Figure 2).

With three pathological phase analysis parameters as the criterion for dyssynchrony, SPECT identified 36 patients with dyssynchrony based on published QGS reference values as described in the methods section. PET identified 33 patients with dyssynchrony based on the same SPECT reference values. With SPECT as a reference standard, PET showed a sensitivity of 64%, a specificity of 82%, a positive predictive value of 70%, and a negative predictive value of 78% (Table 3). SPECT and PET only showed a moderate agreement (kappa .47).

**Table 3:** Contingency table comparing PET to SPECT as standard of reference, criteria for dyssynchrony were three pathological phase analysis parameters.

	SPECT dyssync	SPECT sync	sum
PET dyssync	23	10	33
PET sync	13	47	60
Sum	36	57	93

The calculated sensitivity was 64%, the specificity was 82%, PPV 70%, NPV 78%.

**Figure 3:**
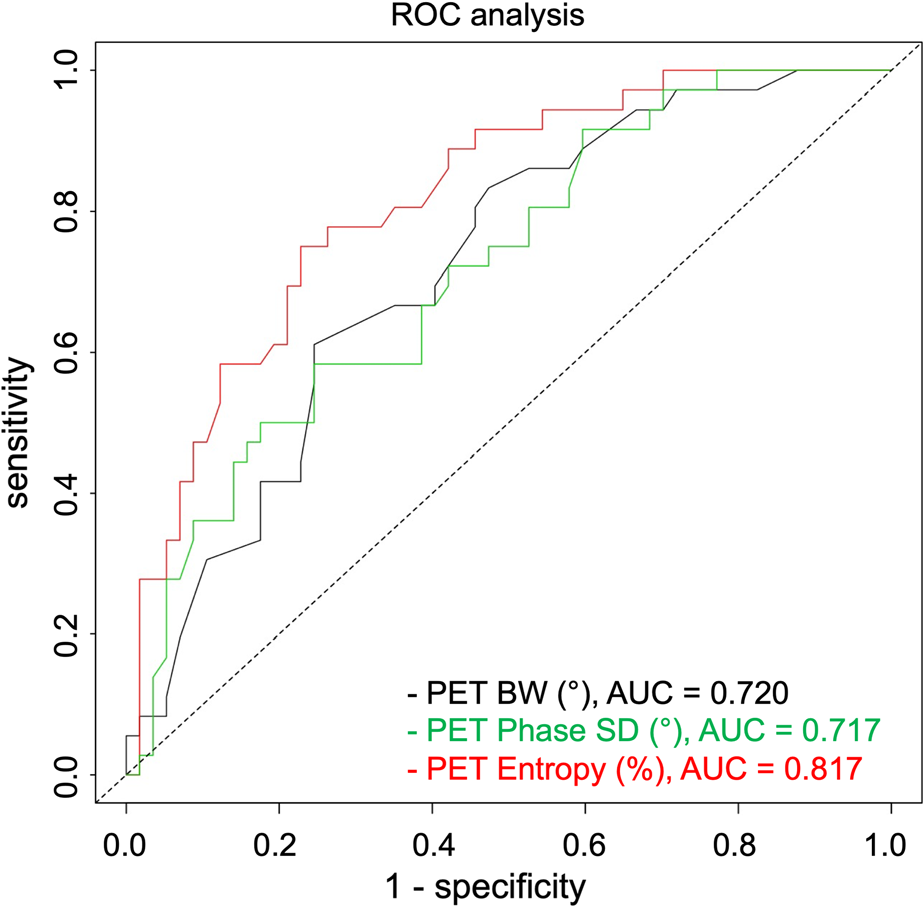
ROC analysis for PET BW, Phase SD, and Entropy to predict dyssynchrony (criterion for dyssynchrony: three pathological phase analysis parameters)

ROC analysis (Figure 3) revealed that the best PET parameter to predict dyssynchrony is Entropy (AUC = .817). BW and Phase SD showed a slightly inferior performance that did not reach statistical significance (AUC = .721 and .717, respectively, *P* = ns in all comparisons).

Cut-off values for single parameters optimized for sensitivity and specificity using Youden’s *J* statistic were 126° for PET BW (sensitivity 61%, specificity 75%, PPV 61%, NPV 75%), 39° for PET Phase SD (sensitivity 50%, specificity 83%, PPV 64%, NPV 72%), and 63% for PET Entropy (sensitivity 69%, specificity 77%, PPV 66%, NPV 80%).

With two pathological phase analysis parameters as the criterion for dyssynchrony, SPECT identified 46 patients with dyssynchrony based on published QGS reference values as described in the methods section. PET identified 54 patients with dyssynchrony based on the same SPECT reference values. With SPECT as a reference standard, PET showed a sensitivity of 74%, a specificity of 57%, a positive predictive value of 63%, and a negative predictive value of 69% (Table 4). SPECT and PET only showed a fair agreement (kappa .31).

**Table 4:** Contingency table comparing PET to SPECT as standard of reference, criteria for dyssynchrony were two pathological phase analysis parameters

	SPECT dyssync	SPECT sync	Sum
PET dyssync	34	20	54
PET sync	12	27	39
Sum	46	47	93

The calculated sensitivity was 74%, the specificity was 57%, PPV 63%, NPV 69%.

**Figure 4:**
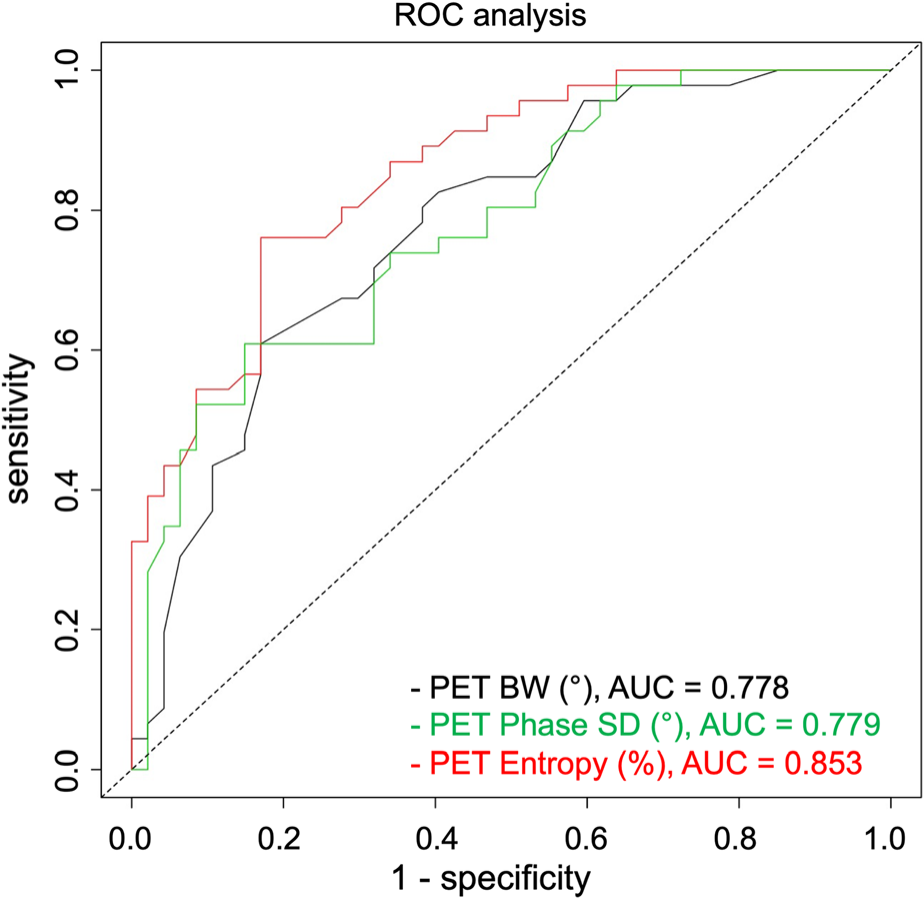
ROC analysis for PET BW, Phase SD, and Entropy to predict dyssynchrony (criterion for dyssynchrony: two pathological phase analysis parameters)

ROC analysis (Figure 4) revealed that the best PET parameter to predict dyssynchrony is Entropy (AUC = .853). BW and Phase SD showed a slightly inferior performance, and the difference did not reach statistical significance (AUC = .778 and .779, respectively, *P* = ns in all comparisons).

Cut-off values for single parameter optimized for sensitivity and specificity using Youden’s *J* statistic were 126° for PET BW (sensitivity 61%, specificity 83%, PPV 78%, NPV 68%), 33° for PET Phase SD (sensitivity 61%, specificity 85%, PPV 80%, NPV 69%), and 62% for PET Entropy (sensitivity 76%, specificity 83%, PPV 81%, NPV 78%).

## Discussion

Phase analysis using the QGS software package was feasible in all gated SPECT and PET datasets and yielded satisfactory results.

The parameters BW and Phase SD differed significantly between the SPECT and PET datasets and only showed a moderate linear correlation between the methods.

There was no significant difference in Entropy between the SPECT and PET datasets and the parameter showed a high linear correlation between the methods.

All parameters showed satisfactory agreement in the Bland–Altman plots.

With gated SPECT as a reference standard, the diagnostic performance of gated FDG PET proved to be limited with only a moderate agreement between SPECT and PET to detect dyssynchrony on a per patient basis.

ROC revealed that Entropy was the best single PET parameter to predict dyssynchrony overall with a cut-off point at 62% to optimize for sensitivity and specificity.

Retrospective phase analysis could be carried out in all gated MPS and gated PET datasets included in the study and yielded satisfactory results. This is facilitated by the fact that phase analysis in modern software packages is a mostly automatic process that requires little to no interaction by the user and has a high intra- and inter-observer reproducibility.^1^ Our experience was in line with published literature and phase analysis parameters could be extracted from all datasets without any problems.

While Pazhenkottil et al. reported no significant differences between gated MPS and gated FDG PET with regard to BW and Phase SD using the Emory Cardiac Toolbox and found a very high linear correlation of those parameters by SPECT and PET,^6^ we could not reproduce these results in our study. BW and Phase SD differed significantly between gated MPS and gated FDG PET datasets and showed only a moderate linear correlation. In contrast to BW and Phase SD, Entropy proved to be superior, yielding no significant difference between both methods, and showed a high linear correlation between SPECT and PET. This is very much in line with the results of a study published in 2020 by Tian et al.,^8^ which found the same moderate linear correlations of BW and Phase SD between SPECT and PET as we did as well as a high linear correlation of Entropy.

Partly, the different results of the Pazhenkottil study might be explained by the use of a different software package. It could be demonstrated in previous investigations that different software packages yield different results and thus cannot be used interchangeably.^10^,^11^

In addition to the aforementioned use of different software packages, there are several other confounders of phase analysis that might hamper the comparability not only between studies but also between different patient cohorts.

A systematic review from 2019^4^ found a wide variety of normal values in patients with no structural heart disease. Apart from software specific characteristics, other confounding factors were identified, such as age, scanner characteristics, and biophysical profile of the study population or cardiovascular risk factors. A 2012 study by Aljaroudi et al.^12^ identified left ventricular function, perfusion defect size, atrial fibrillation, and BMI as additional factors that influence LVMD.

Especially the extent of the myocardial scar tissue seems to be an important factor that is associated with inconsistencies in the evaluation of Phase SD and Entropy.^8^ The purported mechanism here is regional count variations.

As such, patient selection might play a crucial role in the expected outcome of phase analysis, and a comparison between our study and the aforementioned studies can only be made with caution, as crucial differences in patient selection are to be expected.

This is also reflected by the fact that in the Pazhenkottil study mean BW was 168.7° and mean Phase SD was 52.7° measured by SPECT as compared to 94° and 26° in our study, respectively, representing a completely different range of LVMD. Also in the Pazhenkottil study, more than half of the patients were identified with severe dyssynchrony, while in our study only approximately one-third of the patients had dyssynchrony at all.

Method-specific characteristics also seem to play an important role: it seems prudent to assume that while gated SPECT and gated PET should generally detect comparable amounts of dyssynchrony, they represent inherently different methods that will invariably lead to differing measurements of the same variable. Especially PET is enjoying several major advantages with regard to increased count rate and highly improved spatial resolution.^3^ As such, differing results of the phase analysis parameters were to be expected. A more detailed look at the mean values of BW, Phase SD, and Entropy (Table 1) reveals that even though the difference reached statistical significance, the absolute difference is relatively small: approximately 10° for BW and 4° for Phase SD.

The moderate linear correlations of BW and Phase SD, however, suggest that gated MPS and gated FDG PET scans should not be used interchangeably for the repeated measurements of dyssynchrony, especially for serial therapy monitoring. For the lack of an independent gold standard, it is not possible to determine, which of the methods delivers the more accurate results. This will have to be investigated in future studies with external reference standards.

In summary, phase analysis for the assessment of LVMD (irrespective of the method used) is a complex process, as the results are influenced by a plethora of factors, some of which are impossible to eliminate (e.g., TPD, Mismatch and Scar influence-measured LVMD, see Supplement). Nevertheless, to minimize the influence of those factors, it seems advisable to adhere to standardized imaging protocols, to not use the methods interchangeably on follow-up examinations and to have a clear idea of what problems are to be expected in diverse patient populations.

Based on the previously published reference values for gated MPS studies analyzed with QGS^5^ and the three-parameter criterion for dyssynchrony, the SPECT method found 36 patients with LVMD. Gated FDG PET yielded 33 patients with dyssynchrony based on the same SPECT reference values. However, at a kappa of only .47 the agreement between the methods was only moderate. This is again in stark contrast to the findings of Pazhenkottil et al., who found an agreement of the methods of 93% based on SPECT cut-off values for BW and Phase SD.^6^

Again, our results are more in line with the findings of Tian et al. that detected a low agreement between the methods at a kappa of .29.^8^ The better performance in our study might be due to the use of three and not only two parameters to detect dyssynchrony. Furthermore, we based our evaluation on reference values established especially for QGS in healthy individuals, while Tian et al. based their evaluation on cut-off values established for the prediction of CRT response using the Emory Cardiac Toolbox.

When the criterion for dyssynchrony is based on pathological results in only two instead of three of the phase analysis parameters, the sensitivity of PET increases, while the specificity decreases and the agreement between the SPECT and PET methods deteriorates (kappa .31). This was to be expected, since more dyssynchrony is detected by PET, but not necessarily in the same patients as by the standard of reference SPECT.

One problem that might explain the limited diagnostic performance of gated FDG PET in the detection of LVMD is the lack of a true external gold standard, as the use of gated SPECT as the only reference standard for gated PET is in itself flawed and might be prone to misclassifications. Also gated PET might ultimately prove to be the more accurate method.

Based on the three-parameter criterion, ROC analysis of the gated PET parameters BW, Phase SD, and Entropy revealed that Entropy proved to be the best discriminator between synchronous and dyssynchronous patients as defined by the gated SPECT reference values with an AUC of .817. The optimized cut-off value for Entropy was 63%. The optimized cut-off point for BW was 126° and 39° for Phase SD. These cut-off points were very different from those found in the 2011 and 2012 PET studies by Cooke and Aljaroudi.^3^ However, this is most likely explained by the different tracer (Rubidium) and the different software packages (4DM SPECT and Emory Cardiac Toolbox) used in these studies.

Interestingly, when the SPECT reference standard for dyssynchrony was based on the two-parameter criterion, the discrimination of synchronous and dyssynchronous patients by PET actually improved somewhat, with Entropy being the best parameter with an optimized cut-off value of 62% and an AUC of .853.

A possible explanation would be the increased sensitivity of the reference standard for the detection of dyssynchrony and subsequently a higher chance of PET to differentiate between the two patient cohorts.

In the end, it is a striking demonstration that the diagnostic performance of any method aiming to detect LVMD, is also dependent on the definition of LVMD itself.

To objectively determine, whether LVMD is present, and to quantify it, will be one of the ultimate challenges of phase analysis.

## New Knowledge Gained

In contrast to some of the previously published data, we could demonstrate that the agreement between the gated SPECT and gated PET-based phase analysis is not optimal and that the methods cannot simply be used interchangeably, especially for serial imaging and therapy monitoring. Based on reference values for BW, Phase SD, and Entropy specifically established for the QGS software package, we used gated MPS as a reference standard to evaluate the diagnostic performance of gated FDG PET and were able to identify Entropy as the best predictor of dyssynchrony with an optimized cut-off point of 63%.

## Conclusion

Both gated MPS and gated FDG PET are promising and valuable tools for the assessment of LVMD. However, the agreement of both methods at the present time is limited and they cannot be used interchangeably without further modification. Establishing reference ranges and cut-off values is difficult due to the lack of an external gold standard. Further prospective research will be necessary as to which approach will prove more reliable and accurate in the long term, even though PET seems to have an advantage due to the better count statistics and superior spatial resolution.

## Limitations

There are several limitations to our study, the reader should be aware of.

First and foremost, it is a retrospective study and as such the data will have to be validated in further prospective research and in larger patient cohorts.

Gated MPS was used as a reference standard for the evaluation of the diagnostic performance of FDG PET. We elected to proceed this way for the lack of a better external gold standard and since published QGS reference values were available only for gated MPS. Of course, this approach in itself is flawed, since gated FDG PET might prove to be the more accurate method. However, this way it was possible to conduct an analysis of gated FDG PET performance based on objective criteria for dyssynchrony instead of relying on visual assessment. Thus, we could deliver preliminary data to give more insight into which PET parameters might be especially useful for assessing dyssynchrony.

Finally, we elected to assign only those patients to the dyssynchrony cohort that simultaneously showed pathological values for BW, Phase SD, and Entropy (three-parameter criterion) or pathological results for at least two of those parameters (two-parameter criterion). Of course, it could be argued that less severe cases of LVMD might not result in as many parameters reaching pathological levels. But for the sake of clarity our approach seemed reasonable.

## Supplementary Information

Below is the link to the electronic supplementary material.

Supplementary file1 (DOCX 36 kb)

Supplementary file2 (PPTX 1657 kb)

Supplementary file3 (M4A 651 kb)

## Abbreviations

AUC: Area under the curve
BW: Bandwidth
CRT: Cardiac resynchronization therapy
FDG: ^18^F-fluorodeoxyglucose
HF: Heart failure
LVMD: Left ventricular mechanical dyssynchrony
MPS: Myocardial perfusion SPECT
PET: Positron emission computed tomography
Phase SD: Phase standard deviation
SPECT: Single-photon emission computed tomography
