# Supplementary material for: The assessment of left ventricular mechanical dyssynchrony from gated 99mTc-tetrofosmin SPECT and gated 18F-FDG PET by QGS: a comparative study
Source: J Nucl Cardiol. 2021 Jul 19;29(5):2350–60. doi: 10.1007/s12350-021-02737-0 (PMC9553767; doi:10.1007/s12350-021-02737-0)
Supplement: Supplementary file 1 — Supplementary file1 (DOCX 36 kb) [file 12350_2021_2737_MOESM1_ESM.docx]

**Supplement**

Data on total perfusion deficit (TPD), Mismatch (hibernating myocardium) and Scar, were available for 91 of the 93 patients included in the study.

Of the 56 patients that were assigned to the synchrony cohort by gated MPS 55 patients (98%) had a perfusion deficit, 48 patients (86%) had a mismatch (i.e. partially preserved FDG-uptake, hibernating myocardium) and 55 patients (98%) had a scar.

Of the 35 patients that were assigned to the dyssynchrony cohort by gated MPS, 35 patients (100%) had a perfusion deficit, 33 patients (94%) had a mismatch and 35 patients (100%) had a scar.

In the synchrony cohort mean TPD was 16 ± 12%, Mismatch was 4.3 ± 4.4% and Scar was 12 ± 10%.

In the dyssynchrony cohort mean TPD was 33 ± 15%, Mismatch was 9.5 ± 7.4% and Scar was 23 ± 14%.

The differences between synchronous and dyssynchronous patients were statistically significant for all three parameters (p<0.001 in all groups).

In the entire cohort, there was a positive correlation between TPD, Mismatch and Scar and the SPECT and PET phase analysis parameters, indicating increasing amounts of detected LVMD with increasing amounts of perfusion defects, hibernating myocardium and scar. The correlation was weakest for Mismatch, partly not reaching statistical significance in PET, maybe due to the fact that only small amounts of hibernating myocardium were present.

The correlations are summarized in the following Table:

|  | **SPECT BW (°)** | | **SPECT Phase SD (°)** | | **SPECT Entropy (%)** | |
| --- | --- | --- | --- | --- | --- | --- |
|  | **R** | **p** | **R** | **p** | **R** | **p** |
| **TPD (%)** | 0.52 | <0.001 | 0.51 | <0.001 | 0.55 | <0.001 |
| **Mismatch (%)** | 0.27 | 0.009 | 0.23 | 0.0028 | 0.35 | <0.001 |
| **Scar (%)** | 0.48 | <0.001 | 0.49 | <0.001 | 0.48 | <0.001 |
|  | **PET BW (°)** | | **PET Phase SD (°)** | | **PET Entropy (%)** | |
|  | **R** | **p** | **R** | **p** | **R** | **p** |
| **TPD (%)** | 0.49 | <0.001 | 0.45 | <0.001 | 0.48 | <0.001 |
| **Mismatch (%)** | 0.17 | 0.105 | 0.16 | 0.134 | 0.23 | 0.029 |
| **Scar (%)** | 0.48 | <0.001 | 0.44 | <0.001 | 0.45 | <0.001 |

As such, our findings are basically consistent with recent literature (1-3), namely that the extent of LVMD detected by SPECT and PET is influenced by the presence of perfusion defects, hibernating myocardium and myocardial scarring.

**References**

(1) Tian Y, Zhao M, Li W, Zhu Z, Mi H, Li X et al. Left ventricular mechanical dyssynchrony analzyed by Tc-99m sestamibi SPECT and F-18 FDG PET in patients with ischemic cardiomyopathy and the prognostic value. Int J Cardiovasc Imaging 2020;36:2063-71.

(2) Aljaroudi W, Alraies MC, Brunken R, Cerquiera M, Jaber WA. Paradoxical septal motion from prior coronary artery bypass graft surgery does not impact left ventricular mechanical dyssynchrony by gated myocardial perfusion imaging. J Nucl Cardiol 2012;19:1190-7.

(3) Wang L, Wei HX, Yang MF, Guo J, Wang JF, Fang W et al. Phase analysis by gated F-18 FDG PET/CT for left ventricular dyssynchrony assessment: a comparison with gated Tc-99m sestamibi SPECT. Ann Nucl Med 2013;27:325-34.
