# Supplementary material for: The assessment of left ventricular mechanical dyssynchrony from gated 99mTc-tetrofosmin SPECT and gated 18F-FDG PET by QGS: a comparative study
Source: J Nucl Cardiol. 2021 Jul 19;29(5):2350–60. doi: 10.1007/s12350-021-02737-0 (PMC9553767; doi:10.1007/s12350-021-02737-0)
Supplement: Supplementary file 2 — Supplementary file2 (PPTX 1657 kb) [file 12350_2021_2737_MOESM2_ESM.pptx]

## Slide 1
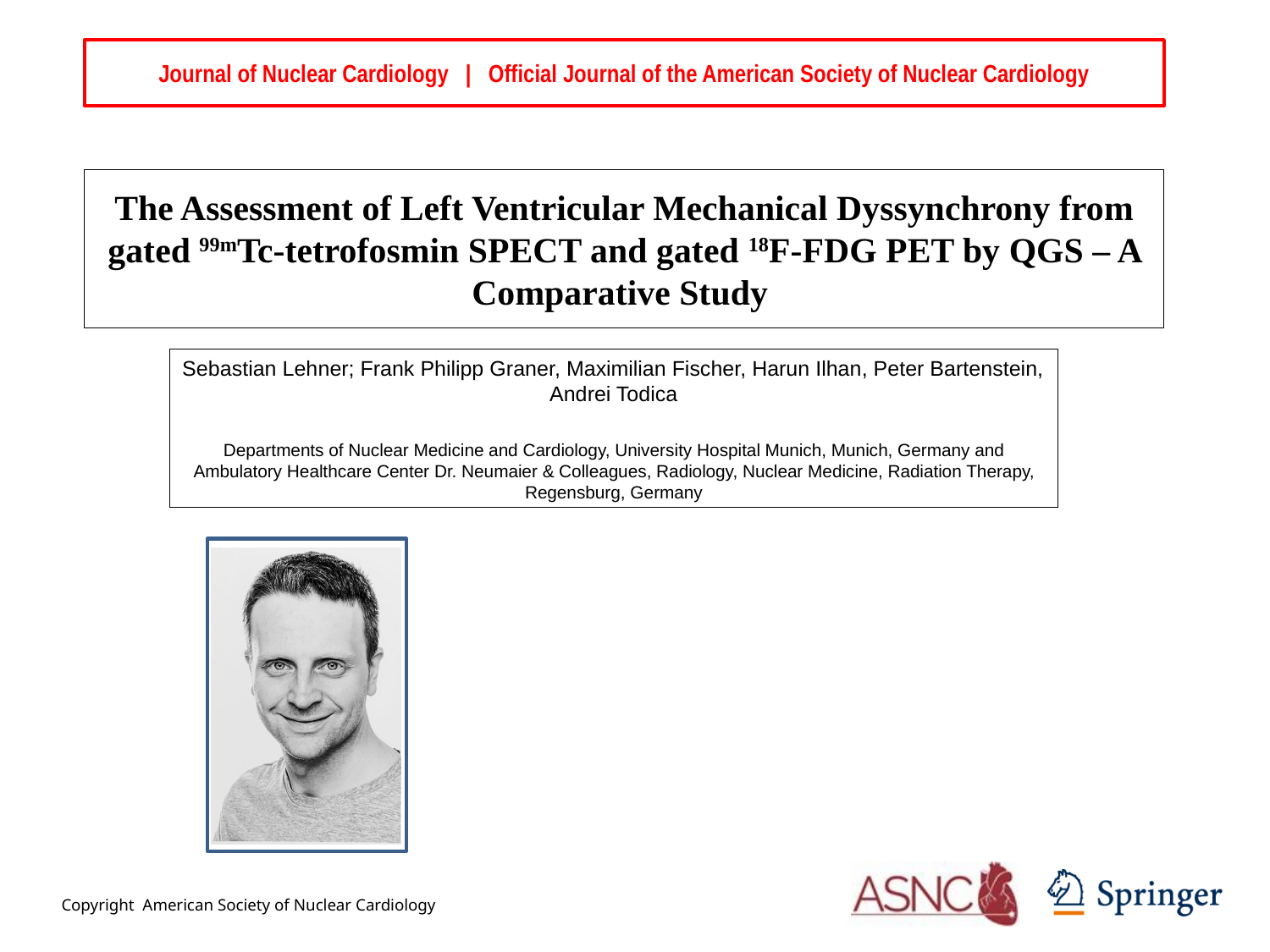

Journal of Nuclear Cardiology | Official Journal of the American Society of Nuclear Cardiology
# The Assessment of Left Ventricular Mechanical Dyssynchrony from gated 99mTc-tetrofosmin SPECT and gated 18F-FDG PET by QGS – A Comparative Study
Sebastian Lehner; Frank Philipp Graner, Maximilian Fischer, Harun Ilhan, Peter Bartenstein, Andrei Todica
Departments of Nuclear Medicine and Cardiology, University Hospital Munich, Munich, Germany and Ambulatory Healthcare Center Dr. Neumaier & Colleagues, Radiology, Nuclear Medicine, Radiation Therapy, Regensburg, Germany
Head shot of author
required
Copyright American Society of Nuclear Cardiology

## Slide 2
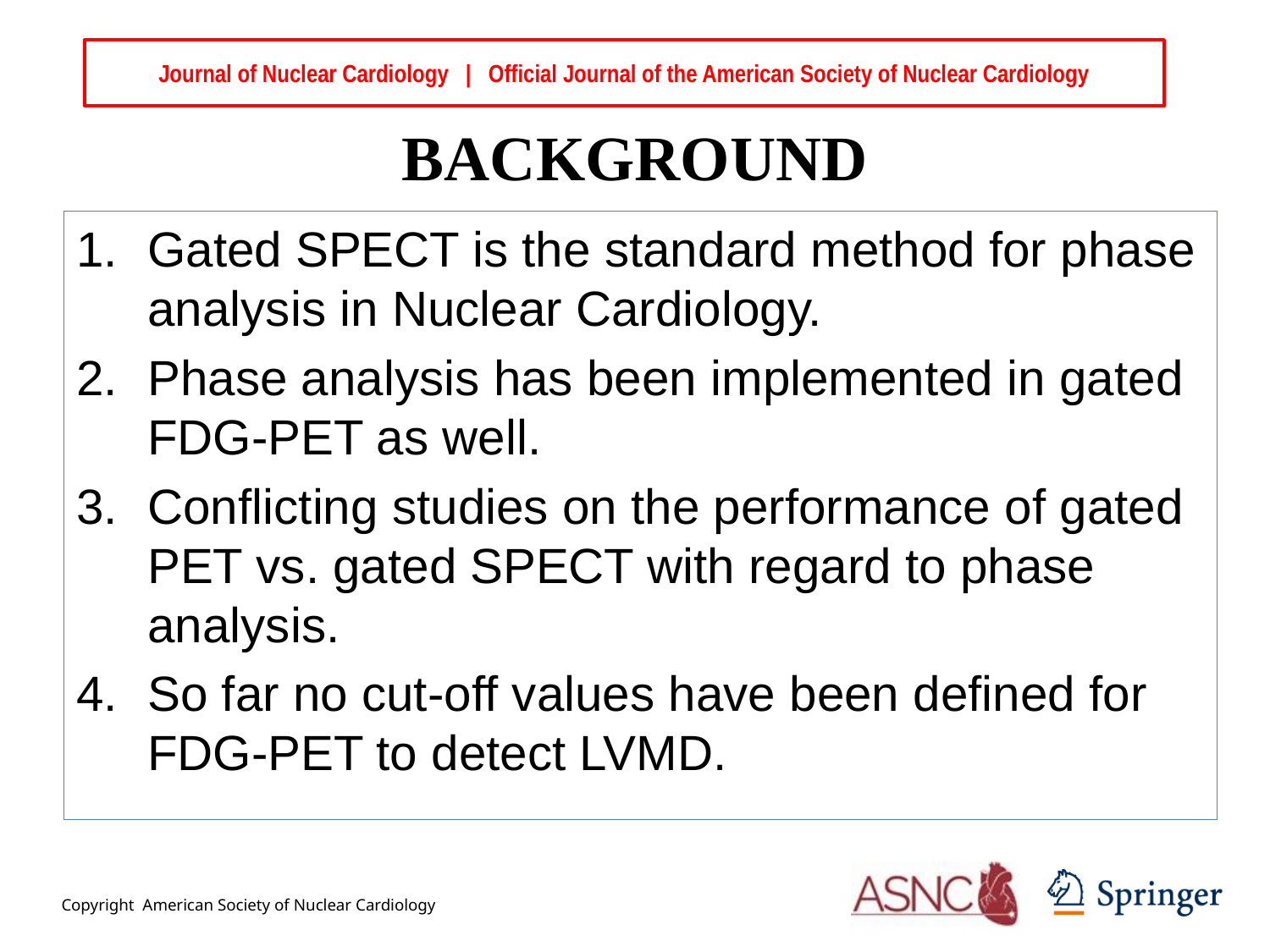

Journal of Nuclear Cardiology | Official Journal of the American Society of Nuclear Cardiology
# BACKGROUND
Gated SPECT is the standard method for phase analysis in Nuclear Cardiology.
Phase analysis has been implemented in gated FDG-PET as well.
Conflicting studies on the performance of gated PET vs. gated SPECT with regard to phase analysis.
So far no cut-off values have been defined for FDG-PET to detect LVMD.
Copyright American Society of Nuclear Cardiology

## Slide 3
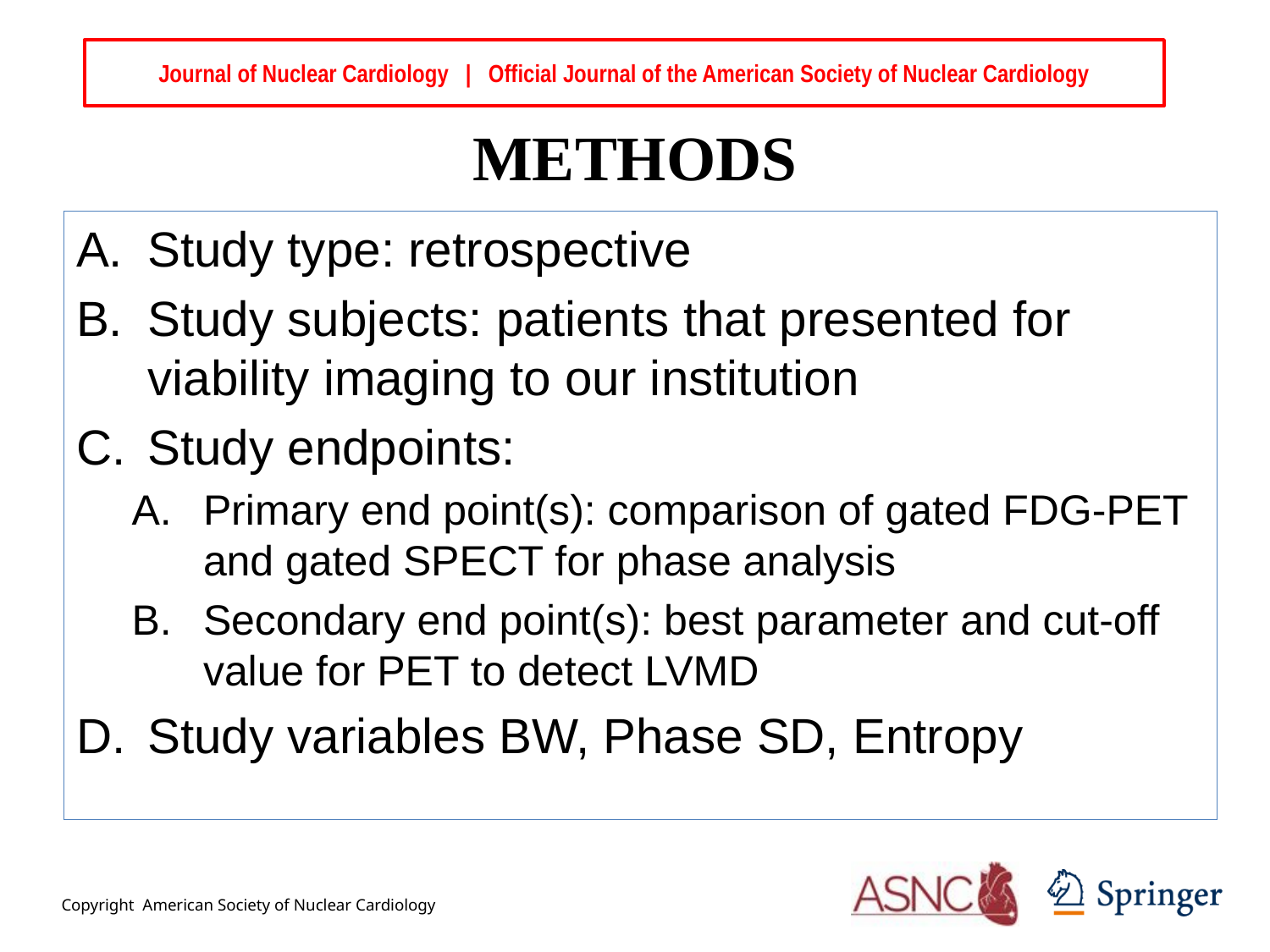

Journal of Nuclear Cardiology | Official Journal of the American Society of Nuclear Cardiology
# METHODS
Study type: retrospective
Study subjects: patients that presented for viability imaging to our institution
Study endpoints:
Primary end point(s): comparison of gated FDG-PET and gated SPECT for phase analysis
Secondary end point(s): best parameter and cut-off value for PET to detect LVMD
Study variables BW, Phase SD, Entropy
Copyright American Society of Nuclear Cardiology

## Slide 4
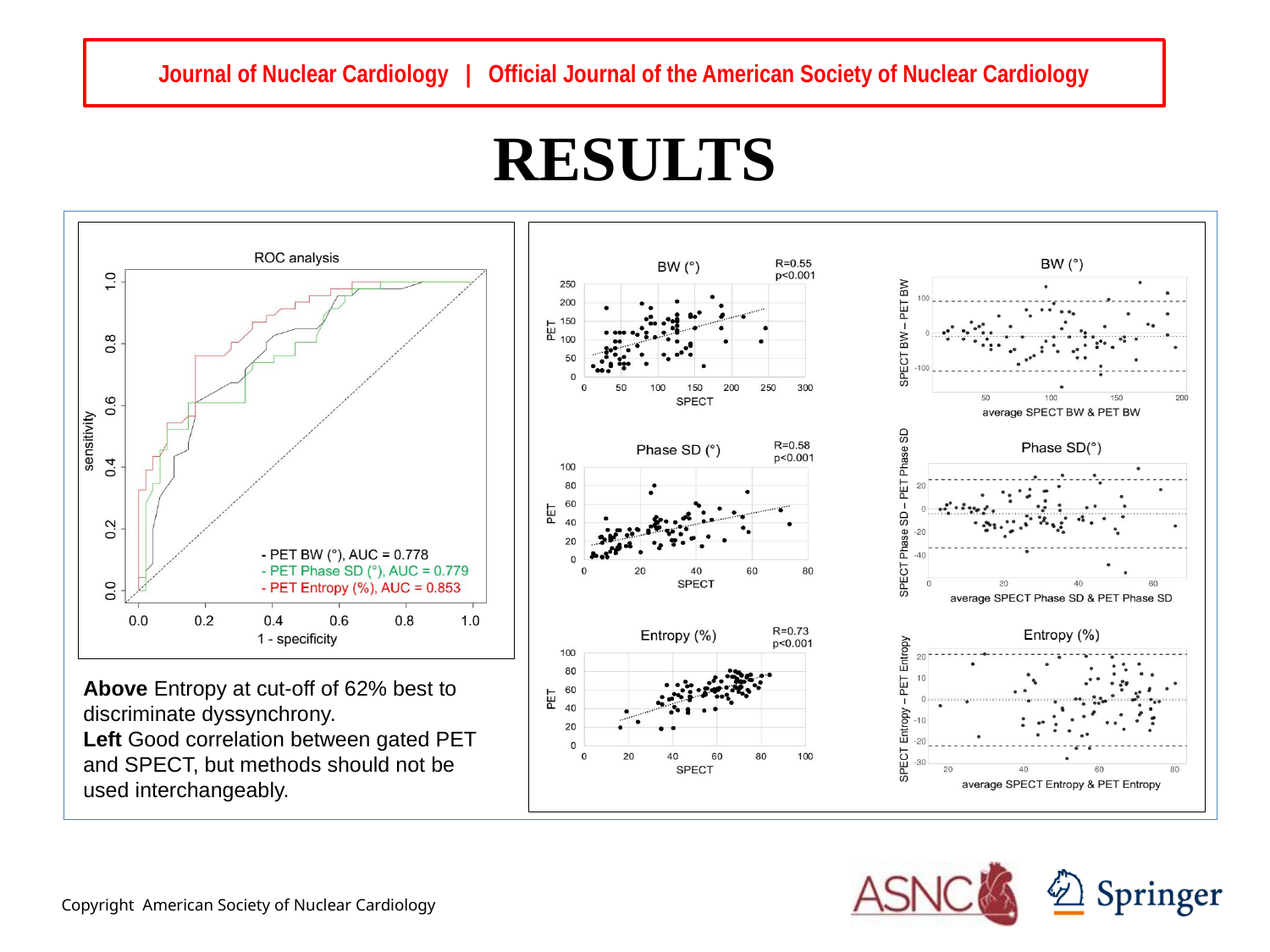

Journal of Nuclear Cardiology | Official Journal of the American Society of Nuclear Cardiology
# RESULTS
Above Entropy at cut-off of 62% best to discriminate dyssynchrony.
Left Good correlation between gated PET and SPECT, but methods should not be used interchangeably.
Copyright American Society of Nuclear Cardiology

## Slide 5
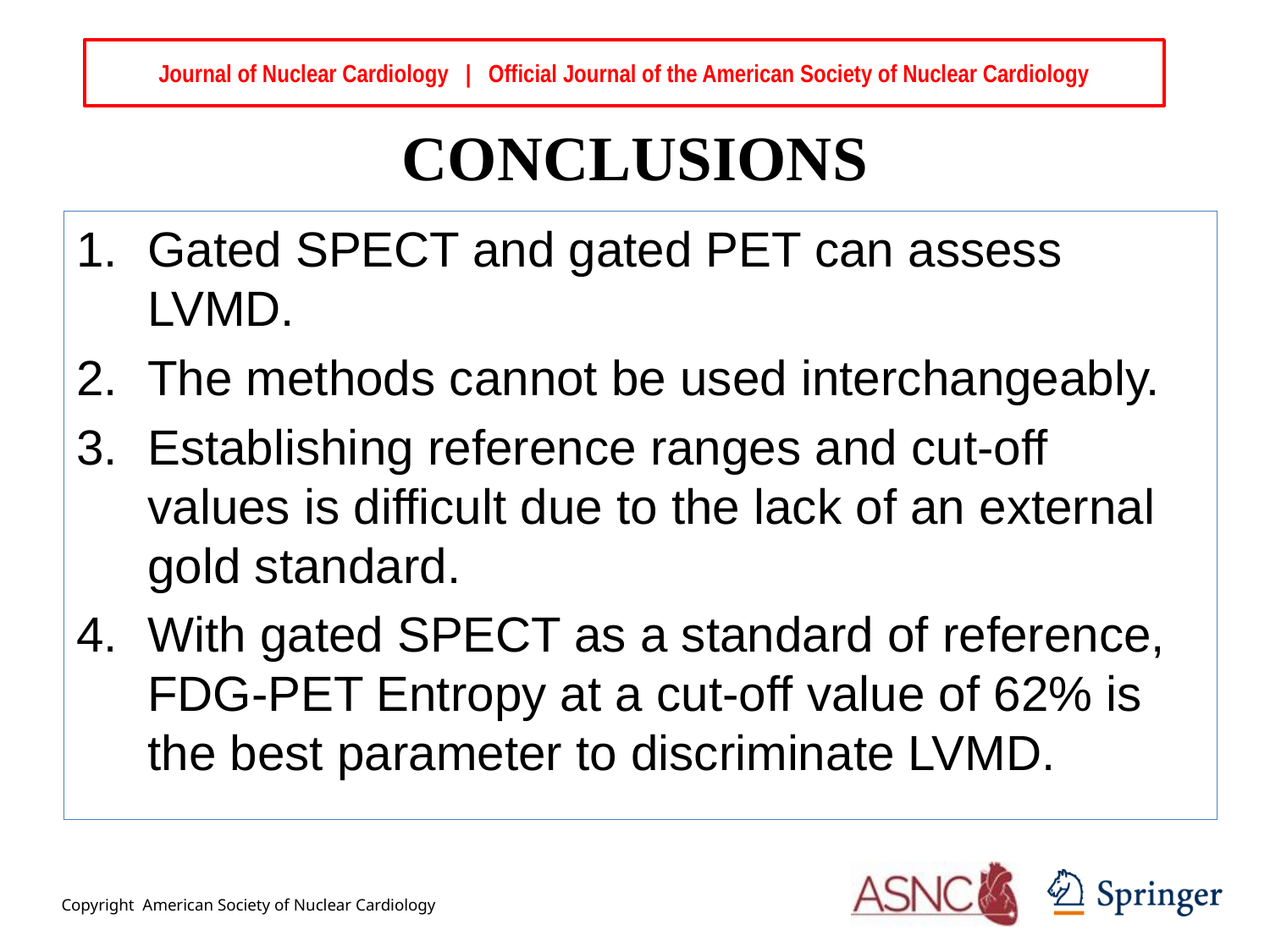

Journal of Nuclear Cardiology | Official Journal of the American Society of Nuclear Cardiology
# CONCLUSIONS
Gated SPECT and gated PET can assess LVMD.
The methods cannot be used interchangeably.
Establishing reference ranges and cut-off values is difficult due to the lack of an external gold standard.
With gated SPECT as a standard of reference, FDG-PET Entropy at a cut-off value of 62% is the best parameter to discriminate LVMD.
Copyright American Society of Nuclear Cardiology
